# Analysis on Salinity Tolerance of Lettuce Cultivars Under Saline Irrigation and Application of Organic Acids

**DOI:** 10.3390/plants14020262

**Published:** 2025-01-17

**Authors:** Jussiara Sonally Jácome Cavalcante, Miguel Ferreira Neto, Tayd Dayvison Custódio Peixoto, Marcondes Pereira da Silva Júnior, Ricardo André Rodrigues Filho, Kariolania Fortunato de Paiva Araújo, Rayane Amaral de Andrade, Lauter Silva Souto, Josinaldo Lopes Araújo Rocha, Luderlândio de Andrade Silva, Pedro Dantas Fernandes, Nildo da Silva Dias, Francisco Vanies da Silva Sá

**Affiliations:** 1 Department of Agronomic and Forest Sciences, Universidade Federal Rural do Semi-Árido, Mossoró 59625-900, RN, Brazil; jussiara_sonally@hotmail.com (J.S.J.C.); miguel@ufersa.edu.br (M.F.N.); marcondesjr20@hotmail.com (M.P.d.S.J.); ricardoarf100@yahoo.com.br (R.A.R.F.); kariolaniafortunato@gmail.com (K.F.d.P.A.); rayane_agronomia@hotmail.com (R.A.d.A.); nildo@ufersa.edu.br (N.d.S.D.); 2 Center of Agrarian and Biological Sciences, Universidade Estadual Vale do Acaraú, São Benedito 62370-000, CE, Brazil; tayd_custodio@uvanet.br; 3 Academic Unit of Agricultural Engineering, Universidade Federal de Campina Grande, Campina Grande 58429-900, PB, Brazil; lauter@ccta.ufcg.edu.br (L.S.S.); josinaldo.lopes@professor.ufcg.edu.br (J.L.A.R.); luderlandioandrade@gmail.com (L.d.A.S.); pedrodantasfernandes@gmail.com (P.D.F.); 4 Department of Agrarian and Exact, Universidade Estadual da Paraíba, Catolé do Rocha 58884-000, PB, Brazil

**Keywords:** *Lactuca Sativa* L., saline stress, leaf gas exchange, chlorophyll a fluorescence, ion homeostasis

## Abstract

Freshwater depletion becomes a significant challenge as the population grows and food demand rises. We evaluated the responses of lettuce cultivars (*Lactuca Sativa*) under saline stress in photosynthetic responses, production, and ion homeostasis. We used a randomized block design in a 3 × 5 factorial scheme with five replications—the first factor: three cultivars of curly lettuce: SVR 2005, Simpson, and Grand Rapids. The second factor consisted of five treatments: T1—control (water of 0.53 dS m^−1^); T2—saline stress (water of 4.0 dS m^−1^); T3—saline stress + ascorbic acid; T4—saline stress + gibberellic acid; and T5—saline stress + salicylic acid. The Grand Rapids lettuce cultivar tolerated water salinity, obtaining the highest production. The Simpson lettuce cultivar was sensitive to salinity, reducing biomass production under saline stress by 11.47% compared to Grand Rapids. Salicylic acid was more effective at mitigating saline stress in the Simpson lettuce cultivar than ascorbic and gibberellic acids, with a 24.85% increase in production compared to saline stress. The findings suggest that the Grand Rapids lettuce cultivar is more resilient to saline conditions, while salicylic acid can significantly enhance production in the sensitive Simpson cultivar under saline stress.

## 1. Introduction

The need to meet the increasing food demand and the growing degradation of water supply promoting the scarcity of water resources is a global concern. Water limitation can be related to environmental or anthropogenic factors, such as droughts, inappropriate use of natural resources, or pollution [1,2]. In regions with limited water resources, crop irrigation can be a major challenge. In such areas, alternative sources of water for crop irrigation play a vital role in ensuring sustainable agriculture. These alternative sources can include treated sewage effluent, fish farm effluent, reject brine from desalination plants, and other sources. These sources offer a unique opportunity to provide water and nutrients to crops, while also addressing the challenge of water scarcity [3]. By utilizing alternative water sources for irrigation, sustainable agriculture practices can be promoted, ensuring food security for communities in water-scarce regions [4].

In the Mossoró/Açu agricultural pole region, Rio Grande do Norte, Brazil, the higher demand for water for irrigation has forced the use of waters with higher salinity levels. Farms in this region demand substantial amounts of water, which has driven the use of saline water, generally between 2.0 and 6.0 dS m^−1^ [5]. However, salt stress promotes adverse effects on plant species’ morphophysiology, metabolic, and respiratory activities. The literature reports that plants may experience various effects due to reduced osmotic potential, ion toxicity, water deficit, stomatal closure, limitations in CO_2_ assimilation, and changes in the photochemical process, which may alter growth and production [5,6,7,8].

Lettuce (*Lactuca sativa* L.) is considered a moderately sensitive crop to salinity, with threshold salinity ranging from 1.3 dS m^−1^ to 1.75 dS m^−1^ [9,10]. Originally from the Mediterranean, lettuce has adapted to the climatic conditions in Brazil. It is accepted for its good palatability, being rich in mineral salts, vitamins, and phytochemicals, and being consumed fresh [11,12]. However, the salt concentration in soil solution affects lettuce cultivars, which can limit crop development and production through morphological and physiological changes [13].

The deleterious effects of salt stress on lettuce have been recorded on biomass production due to ion imbalance toxic and osmotic effects [14,15]. The primary response of plants to salt stress is the osmotic effect. This adjustment occurs via changes in the concentration of organic compounds, such as sugars and amino acids, and their accumulation in plant cells, allowing plants to maintain homeostasis and acclimatize to stress [16,17].

The ability to quickly and accurately perceive the stress signal and activate downstream processes in a timely manner is of utmost importance in successfully coping with salt stress [18]. The best-known exogenous elicitors to mitigate the effect of salinity in lettuce are gibberellic acid (GA_3_) and salicylic acid (SA). Gibberellic acid aims to improve plant growth and production and increase tolerance to abiotic stress [13]. Miceli et al. [2] found that supplementation of 10^−6^ M GA_3_ through lettuce nutrient solution can significantly affect these vegetables’ yield, quality, and post-harvest life.

Salicylic acid improves membrane permeability, function, ion and antioxidant uptake, transport, growth, development, and defense responses in plants under stress conditions [19]. The application of 200 ppm salicylic acid in the Great Lakes cultivar of lettuce increased the parameters of plant height, root length, number of leaves, fresh mass, and dry mass by 24.67%, 16.24%, 51.41%, 103.58%, and 85.92%, respectively, compared to salt stress [14].

Ascorbic acid (ASC) is an exogenous elicitor that acts as a low molecular weight water-soluble antioxidant. It plays a vital role in removing reactive oxygen species and maintaining cellular homeostasis during stress conditions [20,21]. Despite providing satisfactory results to mitigate the effects of salinity in plants, there are few studies in lettuce production. Silva et al. [22], studying the exogenous application of ascorbic acid in leaf cabbage plants, observed that the use of ascorbic acid to minimize the effect of salinity in the production of leaf cabbage seedlings is not recommended because when increasing salinity, there was no positive result in the use of ascorbic acid.

We hypothesized that the exogenous application of ascorbic, gibberellic, and salicylic acids can mitigate the harmful effects of salts from saline water (reject brine) on lettuce cultivars. We also hypothesized that the response of each cultivar to these acids varies. Identifying lettuce cultivars tolerant to saline stress can enhance their cultivation in regions with low-quality water. In addition, improving the resilience of lettuce to saline stress with exogenous application of organic acids will favor food production in these regions. Therefore, this study aimed to evaluate the responses of lettuce cultivars (*Lactuca sativa*) under saline stress in photosynthetic responses, production, and ion homeostasis.

## 2. Results

### 2.1. Leaf Gas Exchange and Chlorophyll a Fluorescence

All leaf gas exchange variables showed a significant effect for the interaction between treatments and lettuce cultivars (*p* ≤ 0.001) (Table 1). Saline stress (4.0 dS m^−1^) decreased the stomatal conductance (*gs*) of lettuce cultivars by 26.67%, 35.56%, and 32.56% for SVR 2005, Simpson and Grand Rapids, respectively, when compared to the control (0.53 dS m^−1^) (Table 1). In SVR 2005, exogenous application of ascorbic acid (ASC) and salicylic acid (SA) increased stomatal conductance by 22.72% and 31.82%, respectively, compared to salt stress. In Simpson, exogenous application of ASC and gibberellic acid (GA_3_) increased stomatal conductance by 10.34% and 27.59%, respectively, relative to salt stress. For the cultivar Grand Rapids, all organic acids improved stomatal conductance in relation to salt stress, with increases of 17.24%, 10.34%, and 27.59%, respectively (Table 1). Under saline stress condition, the lowest *gs* value (0.22 mol (H_2_O) m^−2^ s^−1^) was verified in cultivar SVR 2005.

Transpiration (*E*) was decreased by 20.06%, 25.58%, and 26.25% in the treatment with saline stress (4.0 dS m^−1^) for SVR 2005, Simpson and Grand Rapids, respectively, when compared to the control (0.53 dS m^−1^) (Table 1). In SVR 2005, exogenous application of ASC and SA increased *E* by 18.92% and 27.03%, respectively, relative to saline stress. In Simpson, exogenous applications of ASC, GA_3_, and SA increased transpiration by 11.34%, 9.62%, and 13.40% compared to saline stress treatment. For Grand Rapids, the organic acids that improved transpiration in relation to saline stress were ASC and SA, with increases of 26.69% and 28.83%, respectively (Table 1). Under saline stress condition, the lowest *E* value was verified in cultivar SVR 2005 and the highest *E* value in cultivar Simpson (Table 1).

Saline stress decreased the internal concentration of CO_2_ (*Ci*) in the cultivar Simpson, in relation to the control (0.53 dS m^−1^), with decrease of 13.43% (Table 1). Different behavior was observed in the cultivars SVR 2005 and Grand Rapids, not differing between the treatment under saline stress and control. For the Simpson cultivar, all organic acids (ASC, GA_3_, and SA) increased *Ci* by 15.95%, 6.47%, and 9.05% in relation to saline stress treatment, respectively (Table 1). Under saline stress condition, the lowest *Ci* value was verified in the Simpson cultivar and the highest in the SVR 2005 cultivar (Table 1).

The assimilation rate of CO_2_ (*A_N_*) of the cultivars SVR 2005, Simpson, and Grand Rapids was decreased under saline stress (4.0 dS m^−1^), 19.47%, 11,10%, and 18.37%, respectively, in relation to the control. In SVR 2005, exogenous application of ASC and SA increased *A_N_* by 25.94% and 25.02%, respectively, compared to saline stress. In Simpson, the application of organic acids did not increase the assimilation rate of CO_2_. For Grand Rapids, all organic acids improved the assimilation rate of CO_2_ in relation to saline stress, with increases of 19.21% (ASC), 17.59% (GA_3_), and 21.66% (SA) (Table 1). Under saline stress conditions, the lowest *A_N_* was found in the SVR 2005 cultivar and the highest in Simpson (Table 1).

Saline stress increased the instantaneous water use efficiency (*WUE*) of lettuce cultivars Simpson and Grand Rapids by 19.41% and 10.61%, respectively, when compared to the control (Table 1). The application of GA_3_ increased 14.14% in relation to saline stress in the cultivar Grands Rapids. Under saline stress condition, the lowest *WUE* was verified in SVR 2005 cultivar and the highest in Simpson (Table 1).

There was a decrease in instantaneous carboxylation efficiency (*A_N_*/*Ci*) in SVR 2005 and Grand Rapids cultivars of 20.63% and 14.10%, respectively, under saline stress condition (4.0 dS m^−1^), compared to the control (0.53 dS m^−1^). In lettuce cultivar SVR 2005, exogenous application of organic acids ASC and SA increased about 38.00% and 32.00%, respectively. For Grand Rapids, all organic acids improved instantaneous carboxylation efficiency compared to saline stress, with increases of 26.87% (ASC), 26.87% (GA_3_), and 25.37% (SA) (Table 1). Under saline stress conditions, the lowest *A_N_*/*Ci* was observed in the SVR 2005 cultivar and the highest in Simpson (Table 1).

There was an isolated effect (*p* ≤ 0.05) of lettuce cultivar factors and treatments for maximum PSII quantum efficiency (*Fv*/*Fm*) (Table 2). There was an isolated effect of treatments for quantum efficiency of PSII (*Y*) (*p* ≤ 0.001), photochemical extinction coefficient (*qL*) (*p* ≤ 0.05), regulated photochemical extinction quantum yield (*Y_NPQ_*) (*p* ≤ 0.001), and unregulated photochemical extinction quantum yield (*Y_NO_*) (*p* ≤ 0.001) (Table 2). Minimum fluorescence of illuminated plant tissue (*Fo*’) was observed as an isolated effect of cultivar factor (*p* ≤ 0.05) (Table 2).

The maximum PSII quantum efficiency (*Fv*/*Fm*) showed a significant difference by the mean test only for the treatments factor, with a difference only between the application of gibberellic and salicylic acids under saline stress, with SA being only 2.09% higher than AG_3_ (Table 2). There was a decrease in PSII quantum efficiency (*Y*) in the treatments with gibberellic acids of 24.25%, compared to the control treatment (0.53 dS m^−1^) (Table 2). In relation to saline stress (4.0 dS m^−1^), there was only a decrease in *Y* (16.64%) when gibberellic acid was used. Regarding minimum fluorescence of illuminated plant tissue (*Fo*’), there was only a difference between the control treatment and the treatment under saline stress, with an increase in *Fo*’ of 24.17% under saline stress (Table 2).

The photochemical extinction coefficient (*qL*) showed a difference between the treatment under saline stress and the treatment with the application of gibberellic acid. There was a decrease in *qL* of about 40.91% in the treatment under GA_3_ application compared to saline stress (Table 2). The regulated photochemical extinction quantum yield (*Y_NPQ_*) showed an increase of 26.69% in the treatment under saline stress compared to the control. Among the treatments under saline stress, the highest *Y_NPQ_* was found in the treatment with AG_3_, with an increase of 25.79% compared to saline stress (Table 2). The unregulated photochemical extinction quantum yield (*Y_NO_*) was highest in the treatment under AG_3_ application, with an increase of 45.83% and 55.55% compared to the control and to the treatment under saline stress, respectively (Table 2).

The interaction between treatments and lettuce cultivars was significant for electron transport rate (*ETR*) and leaf temperature (*Tl*) (*p* ≤ 0.001) (Table 3).

There was an increase in electron transport rate (*ETR*) of 184.91%, 148.35%, and 161.27% for lettuce cultivars SVR 2005, Simpson, and Grand Rapids, respectively, in the treatment with saline stress, when compared to the control (Table 3). The application of salicylic acid caused a higher *ETR* in the Simpson cultivar (63.42), followed by SVR 2005 (39.60) and Grand Rapids (25.56) (Table 3).

Saline stress decreased leaf temperature (*Tl*) by 2.48%, 3.04%, and 1.65% in SVR 2005, Simpson, and Grand Rapids lettuce cultivars, respectively, compared to the control (Table 3). In SVR 2005, exogenous applications of ASC, GA_3_, and SA increased leaf temperature by 3.09%, 3.33%, and 3.51% relative to saline stress. In Simpson, exogenous applications of ASC, GA_3_, and SA increased leaf temperature by 0.72%, 2.29%, and 4.41% compared to saline stress. Grand Rapids followed the same trend: the ASC, GA_3_, and SA increased *Tl* relative to saline stress, with increases of 2.93%, 0.78%, and 1.50%, respectively (Table 3).

### 2.2. Production, Biomass, Tissue Water Content and Leaf Concentration of K^+^ and Na^+^

The interaction between treatments and lettuce cultivars was significant for production (P) and number of leaves (*NL*) (*p* ≤ 0.001), and for shoot dry mass (*SDM*) (*p* ≤ 0.05) (Table 4).

**Figure 1:**
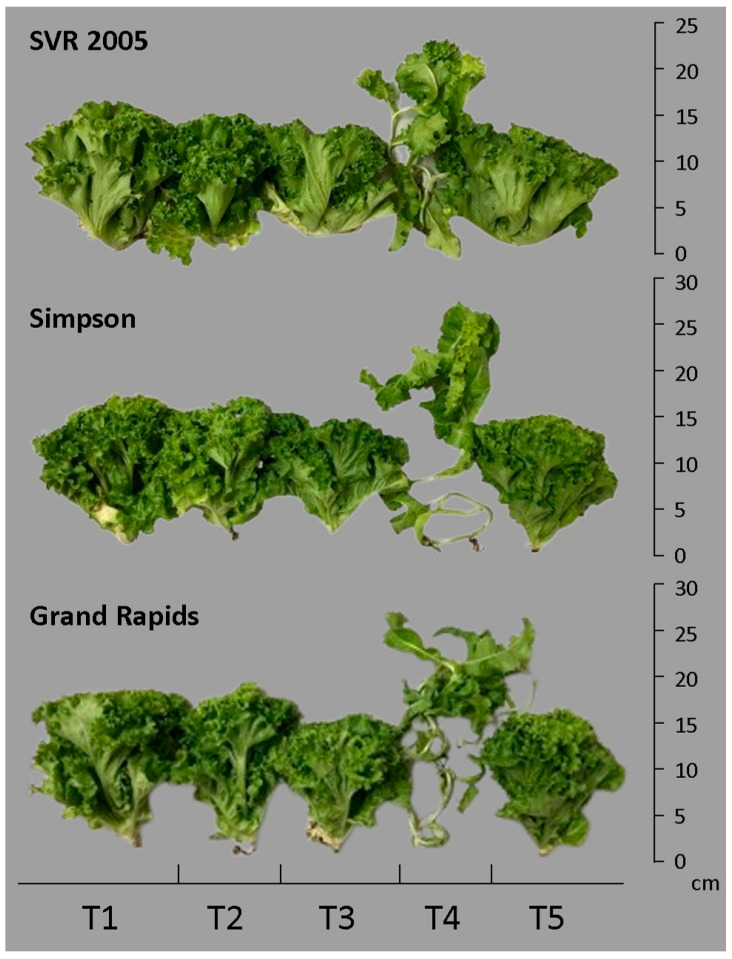
Lettuce cultivars submitted to the treatments. T1—control (0.53 dS m^−1^); T2—saline stress (4.0 dS m^−1^); T3—saline stress + ascorbic acid (50 µmol L^−1^); T4—saline stress + gibberellic acid (50 µmol L^−1^); and T5—saline stress + salicylic acid (50 µmol L^−1^).

Lettuce cultivars that received applications of ascorbic and salicylic acids showed normal growth, but gibberellic acid application caused disturbed growth of lettuce plants (Figure 1). Production (*PROD*) decreased by 29.53% and 34.93% for cultivars SVR 2005 and Simpson, respectively, under saline stress of 4.0 dS m^−1^ and compared to the control (Table 4). In the Simpson cultivar, the application of SA increased production by 24.85%, compared to saline stress, whereas in the cultivars SVR 2005 and Grand Rapids there was no increase by the application of organic acids (Table 4 and Figure 1). Under saline stress conditions, the lowest production was observed in the SVR 2005 and Simpson cultivars and the highest in the Grand Rapids cultivar (Table 4).

Saline stress decreased the number of leaves (*NL*) in the Simpson cultivar by 39.23% compared to the control. In SVR 2005 and Grand Rapids, there were no differences between control and saline stress. In Simpson, ASC and SA increased *NL* by 27.85% and 37.97%, respectively, compared to saline stress. In Grand Rapids, ASC caused a decrease in *NL* of 16.50% compared to the saline stress treatment (Figure 1). The treatment with ASC showed the highest *NL* value (20.20) in the Simpson cultivar, followed by Grand Rapids (17.20) and SVR 2005 (14.60) (Table 4).

There was no statistical difference in shoot dry mass (*SDM*) when comparing saline stress and control for the three cultivars investigated. The application of AG_3_ to lettuce cultivars caused a decrease in *SDM* in all cultivars compared to saline stress, with a decrease of 50.11%, 61.52%, and 37.86% for SVR 2005, Simpson, and Grand Rapids, respectively (Table 4). The lowest *SDM* in the treatment of saline stress was found in cultivar SVR 2005, with Simpson and Grand Rapids higher, but not statistically different.

There was an isolated effect for the factors treatments and cultivars for tissue water content (TWC) (*p* ≤ 0.001) (Table 5). There was no significant effect for the potassium K^+^ content (*p* > 0.05), there was an isolated effect of treatments (*p* ≤ 0.001) and cultivars (*p* ≤ 0.05) on leaf Na^+^ concentration, and, for the sodium/potassium ratio (Na^+^/K^+^), there was an isolated effect of treatments (*p* ≤ 0.001) (Table 5).

The application of ascorbic and gibberellic acid caused a decrease of 1.55% and 1.27%, respectively, in TWC, when compared to the control (Table 5). The cultivar SVR 2005 showed higher TWC compared to the cultivars Simpson and Grand Rapids.

Saline stress increased Na^+^ by 121.86% in relation to the control, and in the treatments with the application of organic acids there was no significant difference in relation to saline stress. Among the cultivars, the cultivar Simpson concentrated more sodium than the cultivar Grand Rapids, but neither differed from SVR 2005. In the Na^+^/K^+^ ratio, there was an increase in saline stress of 107.69% compared to the control (Table 5).

## 3. Discussion

It is vital to find sustainable ways to ensure a reliable water source for agriculture due to increasing food demand. One such approach is to mix fresh water and saline water from desalination plants, which can help address the problem of water scarcity by reducing freshwater use. However, the presence of excess salts in saline water can have a detrimental effect on crop growth and production. Thus, finding effective ways to mitigate the impact of salt concentration of saline water on crops is crucial for sustainable agricultural development. Our objectives were to investigate how the concentration of salts affects lettuce production and to identify which organic acids are capable of mitigating the adverse effects of excess salts on leaf gas exchange and production. We evaluated the response of three lettuce cultivars—SVR 2005, Simpson, and Grand Rapids—to saline stress induced by saline water irrigation under exogenous application of ascorbic, gibberellic, and salicylic acids. According to our research, irrigating with saline water led to an increase in electrical conductivity of the soil extract from 0.58 to 6.10 dS m^−1^. This resulted in a decrease in the number of leaves and production of the Simpson cultivar and number of leaves of the SVR 2005 cultivar due to increased salinity and reduced soil osmotic potential. These factors limit the extraction of water by the roots, leading to higher energy expenditure for maintaining water absorption by the plants [11].

The Simpson cultivar exhibited a greater assimilation rate of CO_2_ under saline stress than the SVR 2005 and Grand Rapids cultivars. However, Simpson production and the number of leaves were lower than the Grand Rapids cultivar under conditions of salt stress. Therefore, the Grand Rapids cultivar was more tolerant to salinity than the Simpson cultivar, which can be considered sensitive to salinity. The cultivar Grand Rapids, through the mechanisms of escape from saline stress, also provided the lowest accumulation of Na^+^ in the leaf, compared to SVR 2005 and Simpson cultivars. This trait not only allowed it to evade the effects of saline stress but also led to improved production. Salinity can harm plants by affecting nutrient and ion activities, leading to osmotic and ionic damage, as well as reduced production and quality [23]. Among the most destructive effects of saline stress is the ionic one, with accumulation of Na^+^ and Cl^−^ ions in plant tissues [24]. The entry of both ions into the cells causes significant ionic imbalance, and excess absorption can cause physiological failures, adversely affect plant growth and development, inhibit enzymes, and create nutritional imbalance [25].

Osmoregulation plays an essential role in the adaptation mechanism of lettuce plants to salinity [26]. In our research, the Na^+^/K^+^ ratio was low (lower than 0.60). Hniličková et al. [26] and Ondrasek et al. [27] also observed a marked decrease in the Na^+^/K^+^ ratio in lettuce. However, a high Na^+^/K^+^ ratio is a determining characteristic of saline stress tolerance. The minimum value of the Na^+^/K^+^ ratio to be considered high is above 0.60 [28]. El-Tayeb [29] found that exogenously applied SA decreased Na^+^ and increased K^+^, Ca^2+^, and P content in the shoot and roots of barley seedlings compared to those not treated with SA under saline stress. High levels of Na^+^ inhibit the absorption of essential ions for plants. It is known that for some crops, Na^+^ can partially replace K^+^. On the other hand, potassium plays a crucial role in osmoregulation, turgor maintenance, and protein synthesis, and it activates more than 50 enzymes [30].

The water maintenance of the plants occurs by the high tissue water content (TWC > 94.0%) in all lettuce cultivars produced under saline stress, indicating that the strategies to maintain the high cell turgidity level used by the plants were efficient. These beneficial mechanisms influenced the Grand Rapids cultivar, showing a similar number of leaves and production under saline stress compared to the control.

Regarding chlorophyll *a* fluorescence parameters, the application of SA promoted the best quantum efficiency of PSII (*Y*) among the acids used, and GA_3_ promoted the highest unregulated photochemical extinction quantum yield (*Y_NO_*) than SA. The quantum efficiency of PSII indicates the photosynthetic performance of plants under saline stress [31]. High values of *Y* indicate an increase in photosynthesis [32].

Phytohormones and biostimulants can improve antioxidant activity and enhance phytohormone responses by upregulating genes involved in phytohormone biosynthesis under saline stress conditions [33,34,35]. Benito et al. [34] found that biostimulants increase yield in lettuce under salt stress conditions by upregulating cytokinin biosynthesis. Melatonin can alleviate salinity’s impact on lettuce plants by improving plant growth, productivity, and water status [35]. Gibberellic acid aims to improve plant growth and production and increase tolerance to abiotic stresses [13]. Our research showed that the application of GA_3_ did not provide production gain and number of leaves, compared to saline stress for all cultivars studied. These results differ from those of Miceli et al. [2] who found that supplementation of 10 µM GA_3_ through a nutrient solution in lettuce can significantly affect these vegetables’ production, quality, and post-harvest life. Supporting this, Rech Filho et al. [36] demonstrated similar outcomes in the presence of GA_3_, revealing consistent elongation in vitro in bromeliads, with maximum elongation observed at 10 µM. Such differences may have been caused by the method of application (foliar) and the amount used (50 µM L^−1^) in our research, which was five times higher than that applied in these cited studies. The exogenous application of GA_3_ caused an increase in *WUE* and *A_N_/Ci* in the Grand Rapids cultivar compared to the other cultivars. Regarding the saline stress treatment, the application of GA_3_ increased *Y_NPQ_* and *Y_NO_*, showing that the cultivars have a greater protective capacity against the adverse effects of saline stress. It was attributed to an increased regulated photochemical extinction quantum yield (*Y_NPQ_*), which dissipates energy as heat through the xanthophyll cycle [37].

Salicylic acid improves lettuce plant growth parameters, plant height, root length, fresh mass, dry mass, and number of leaves under salt stress conditions [14]. We found that adding SA in the Simpson cultivar mitigated the inhibition in plant growth as its application increased production and number of leaves compared to saline stress. Therefore, salicylic acid promotes the increment of lettuce plant production in the Simpson cultivar. The increase in production observed in plants subjected to saline stress and treated with salicylic acid occurs due to water amplification and utilization. Plant hormones play an essential role in plant growth and development and can alleviate the adverse effects of saline stress [19]. Exogenous SA supplementation can positively affect the shoot growth of plants by influencing the internal partitioning of resources [14,38]. In addition, increased mineral nutrient uptake and efficient transport of photoassimilates occur by increased membrane permeability. Previous studies indicate that SA exogenous applications can ameliorate membrane deterioration in plants exposed to saline stress, indicating that SA contributes to maintaining membrane functions [14].

Research on using exogenous ascorbic acid in lettuce production as a mitigator of saline stress remains limited. However, ascorbic acid serves as a low molecular weight, water-soluble antioxidant that plays a role in scavenging reactive oxygen species and regulating cellular homeostasis under saline stress conditions [20,21]. In the Simpson cultivar, the application of ASC led to an increase in the number of leaves compared to saline stress. Regarding the physiological parameters, in the treatment with ASC there was an increase in *gs*, *E*, *Ci*, and *Tl* in relation to saline stress. This fact may be related to the acclimatization mechanism of lettuce plants under saline stress conditions with exogenous application of ASC. Xylia et al. [39], studying the application of ASC in lettuce, found an increase in weight, production, and maintenance of quality parameters.

In summary, when lettuce was grown using rejected brine with an electrical conductivity of 4.0 dS m^−1^ for irrigation, it affected leaf gas exchange variables, chlorophyll a fluorescence, production, and ion balance, regardless of the cultivar studied. However, the Grand Rapids cultivar showed the highest resilience and production under saline-stress conditions, indicating its tolerance to salinity. Conversely, the Simpson cultivar was the most sensitive to salinity, displaying the poorest production performance under saline stress conditions. It is interesting to note that salicylic acid was found to be more effective in mitigating saline stress in a salinity-sensitive cultivar (Simpson) compared to ascorbic and gibberellic acids, despite the adverse effects of salinity. These findings offer valuable insights into the impact of saline stress on lettuce production and the potential use of salicylic acid as a mitigation strategy for sensitive lettuce cultivars.

## 4. Materials and Methods

### 4.1. Location and Characterization of the Experimental Area, Plant Material, and Treatments

The experiment was developed in a greenhouse of the Center of Agrarian Sciences of the Federal Rural University of Semi-Arid (UFERSA) in Mossoró, Rio Grande do Norte, Brazil, from December 2019 to February 2020. The city is in the semi-arid region of northeastern Brazil, with geographic coordinates 5°11′ S 37°20′ W and 18 m altitude. The study was conducted in a locality with a hot semi-arid climate characterized by high temperatures and low humidity levels, identified by Köppen’s classification system code as BSh [40].

The experimental design adopted was randomized blocks in a 3 × 5 factorial scheme with five repetitions. The repetitions had 6 plants, totaling 30 plants per treatment. The first factor corresponded to three cultivars of curly lettuce (*Lactuca Sativa* L.): SVR 2005, Simpson, and Grand Rapids. The lettuce cultivars were submitted to the second factor with five treatments that included foliar application of elicitors: T1—irrigation with low salinity water (0.53 dS m^−1^, control); T2—irrigation with high salinity water (4.0 dS m^−1^, saline stress); T3—saline stress + exogenous application of ascorbic acid (50 µM L^−1^); T4—saline stress + exogenous application of gibberellic acid (50 µM L^−1^); and T5—saline stress + exogenous application of salicylic acid (50 µM L^−1^). The concentrations used in this research were based on studies carried out by Sá et al. [41] and Pereira et al. [42].

**Figure 2:**
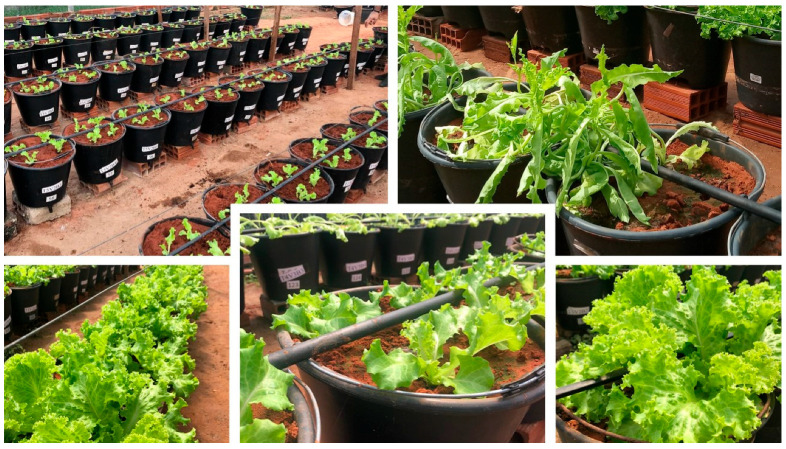
Lettuce Cultivation in greenhouse.

The lettuce seedlings, purchased locally, were transplanted into pots (Figure 2). Foliar applications of organic acids occurred on the 2nd, 10th, and 20th day after transplanting (DAT), with volumes of 2.5, 5.0, and 5.0 mL per plant, totaling 12.5 mL. The soil used in the experiment was collected at the Experimental Farm Rafael Fernandes, located in the rural area of Mossoró, Brazil. It is classified as Ultisol [43] and was taken from a depth of 0–30 cm. Soil samples were air-dried, crushed, and sieved through a 2.0 mm mesh for physical and chemical analysis [44] (Table 6). Each repetition comprised three lysimeters adapted from plastic pots, filled with 12 dm^3^ of soil each. Each pot had its bottom perforated, and a layer of 1.0 dm^3^ of gravel and 2.0 mm nylon mesh was added to assist drainage. Before transplanting the crop, liming was carried out with 5.81 g of calcium hydroxide (Ca(OH)_2_) per pot, containing 54% calcium, aiming to raise base saturation to 90%.

### 4.2. Fertilization and Irrigation Management

Fertilization was conducted according to the guidelines established by Novais et al. [45], specific for pots in greenhouse experiments: 300 mg of P_2_O_5_^−^, 150 mg of K_2_O, and 100 mg of N per dm^3^ of soil using fertigation. The sources were urea (45% N), potassium chloride (KCl = 60% K_2_O), and monoammonium phosphate (MAP = 12% N and 50% P_2_O_5_^−^). This fertilization was divided into three equal parts, the first applied at planting, the second and third applied via fertigation, at 10 and 20 DAT, respectively. Foliar fertilization with micronutrients was performed at 15 DAT with Liqui-Plex Fruit^®^ fertilizer (Alltech, Lexington, KY, USA) at the rate of 3 mL L^−1^ of syrup, following the manufacturer’s recommendation.

Irrigations were carried out every day, early in the morning and late in the afternoon, considering the volume corresponding to the actual evapotranspiration of the crop, measured by drainage lysimetry in additional plots corresponding to the treatments. The characteristics of the raw waters used in the experiment are described in Table 7. A drip irrigation system was used consisting of 16 mm hoses and self-compensating drippers with a flow rate of 1.40 L h^−1^. The irrigation system was composed of a Metalcorte/Eberle circulation pump motor (MetalCorte, Pelotas, Brazil), which was self-ventilated, driven by a single-phase motor, 210 V voltage, 60 Hz frequency, and installed in a 50 L capacity reservoir. The applied volume (AV) per pot was obtained by the difference between the previous depth (PD) applied minus the average drainage (D), divided by the number of containers (n) and leaching fraction (LF) Equation (1).

(1) $$AV=\frac{PD-D}{n\left(1-LF\right)}$$

The total irrigation volume was 12.10 L per pot. In this water volume, 4.10 g of salt were applied to plants irrigated with water supply (0.53 dS m^−1^) and 30.98 g of salts to plants irrigated with saline water (4.0 dS m^−1^). At the end of the experiment, 30 DAT, soil salinity was determined according to the methodology of Richards [46]. The paste saturation extract’s electrical conductivity and pH values are described in Table 8.

### 4.3. Leaf Gas Exchange and Chlorophyll a Fluorescence Determination

Leaf gas exchange was evaluated at 30 DAT from 7 to 11 a.m. The evaluations were conducted on the fully developed leaves, in the upper third of each plant, with the portable infrared gas analyzer (IRGA), model LCPro+ Portable Photosynthesis System^®^ (ADC Bio Scientific Limited, Hoddesdon, UK). The IRGA was programmed for a temperature control at 25 °C, irradiation of 1200 µmol photons m^−2^ s^−1^, and airflow of 200 mL min^−1^. The data collected referred to a set of specific parameters: assimilation rate of CO_2_ (*A_N_*) in µmol m^−2^ s^−1^, transpiration (*E*) in mmol of H_2_O m^−2^ s^−1^, stomatal conductance (*gs*) in mol of H_2_O m^−2^ s^−1^, the internal concentration of CO_2_ (*Ci*) in µmol CO_2_ m^−2^ s^−1^, as well as the leaf temperature (*Tl*) in ºC. With these data, water use efficiency (*WUE*) (*A_N_/E*) in µmol, CO_2_ m^−2^ s^−1^/mmol H_2_O m^−2^ s^−1^, and carboxylation efficiency (*A_N_/Ci*) in decimal were determined [47].

Subsequently, these same leaves were submitted to evaluate chlorophyll *a* fluorescence. The device used was a pulse-modulated fluorometer, model OS5p, from Opti Science. The *Fv*/*Fm* protocol was used for evaluations under dark conditions in which the maximum PSII quantum efficiency (*Fv*/*Fm*) was determined [42]. The evaluations under light conditions, applying the Yield protocol, were to obtain the electron transport rate (*ETR*) and current quantum efficiency of photosystem II (PS II) (*Y*). From these data, the minimum fluorescence of illuminated plant tissue (*Fo*’) [48], photochemical extinction coefficient by the lake model (*qL*), regulated photochemical extinction quantum yield (*Y_NPQ_*), and unregulated photochemical extinction quantum yield (*Y_NO_*) [49] were determined.

### 4.4. Production, Biomass, Tissue Water Content and Leaf Concentration of K^+^ and Na^+^ Determination

After the physiological analysis, the number of leaves of the lettuce plants was determined by simple leaf counting. After counting the leaves on the plants, the shoot was collected to determine production (P) on an analytical balance (0.0001 g). Subsequently, they were packed in Kraft paper bags, placed in an oven with forced air circulation at 65 °C until they were at a constant weight, and weighed to obtain the shoot dry mass (SDM), with the results expressed in g per plant.

Tissue water content (TWC) was determined by comparing the water content of freshly harvested plant tissue (P) with the water content of the same tissue when dried, expressing the result on a percentage basis. The assessment of tissue water content (TWC) involved the comparison between the mass of recently harvested plant tissue (P) and the mass of the corresponding tissue after dehydration (SDM). The result was expressed as a percentage from Equation (2).

(2) $$TWC=\frac{P-SDM}{SDM}\times 100$$

The shoot dry mass was ground using a Willey-type steel mill and stored in labeled plastic bags for subsequent analysis. In the laboratory, the material underwent nitric acid digestion (HNO_3_ 65%) using a microwave oven to produce an extract for determining the total leaf concentrations of potassium (K^+^) and sodium (Na^+^) [50]. The Na^+^/K^+^ ratio was calculated based on the obtained data.

### 4.5. Statistical Analysis

The data underwent analysis of variance and an F test (*p* ≤ 0.05). In cases of significance, isolated factors and the interaction among them were compared using Tukey’s mean comparison test (*p* ≤ 0.05) with the statistical software SISVAR^®^ 5.3 [51].

## 5. Conclusions

The current research findings show that using saline water for irrigation (4.0 dS m^−1^), obtained by mixing reject brine of desalination plants, significantly impacted critical factors for lettuce plant growth. These factors include leaf gas exchange, chlorophyll *a* fluorescence, and ion homeostasis. It was observed that Simpson lettuce cultivar was sensitive to salinity, which significantly reduced production. However, the Grand Rapids cultivar showed remarkable tolerance to water salinity of 4.0 dS m^−1^ and soil salinity of 5.0–6.1 dS m^−1^. Additionally, salicylic acid was more effective in mitigating saline stress in the Simpson cultivar than ascorbic and gibberellic acids. These findings have significant implications for farmers and researchers, highlighting the importance of carefully managing water salinity levels and mitigating substances for optimal plant growth and production.

## Figures and Tables

**Table 1:** Summary of F test and test of means for stomatal conductance (*gs*, in mol (H_2_O) m^−2^ s^−1^), transpiration (*E*, in mmol (H_2_O) m^−2^ s^−1^), internal concentration of CO_2_ (*Ci*, in μmol (CO_2_) mol^−1^), assimilation rate of CO_2_ (*A_N_*, in μmol (CO_2_) m^−2^ s^−1^), instantaneous water use efficiency (*WUE*, in μmol (CO_2_) m^−2^ s^−1^/mmol (H_2_O) m^−2^ s^−1^), and instantaneous carboxylation efficiency (*A_N_*/*Ci*, in μmol (CO_2_) m^−2^ s^−1^/μmol (CO_2_) mol^−1^) of lettuce cultivars irrigated with saline water submitted to exogenous application of organic acids at 30 days after transplanting.

F Test (*p*-Value)							
Variation Sources		*gs*	*E*	*Ci*	*A_N_*	*WUE*	*A_N_/Ci*
Block		0.0016	0.0010	0.9741	0.1274	0.9260	0.5593
Treatments (T)		0.0000	0.0000	0.0000	0.0000	0.0000	0.0028
Cultivars (C)		0.0000	0.0000	0.0000	0.0000	0.0000	0.0000
T × C		0.0000	0.0000	0.0000	0.0000	0.0000	0.0000
CV (%)		3.36	1.96	2.87	4.22	4.57	6.65
Tukey’s Test (Mean ± SE)							
C	T	*gs*	*E*	*Ci*	*A_N_*	*WUE*	*A_N_/Ci*
SVR 2005	T1	0.30 ± 0.004 aB	3.24 ± 0.01 aC	258 ± 0.97 aA	16.23 ± 0.14 aB	5.01 ± 0.03 aAB	0.063 ± 0.001 aB
	T2	0.22 ± 0.002 cB	2.59 ± 0.01 cC	262 ± 1.41 aA	13.07 ± 0.30 bC	5.05 ± 0.10 aC	0.050 ± 0.001 bC
	T3	0.27 ± 0.004 bC	3.08 ± 0.01 bC	240 ± 1.77 bB	16.46 ± 0.13 aB	5.34 ± 0.06 aA	0.069 ± 0.001 aB
	T4	0.22 ± 0.002 cC	2.42 ± 0.02 dC	264 ± 1.66 aA	12.18 ± 0.15 bB	5.03 ± 0.07 aC	0.046 ± 0.001 bC
	T5	0.29 ± 0.010 abB	3.29 ± 0.09 aB	253 ± 1.86 aA	16.34 ± 0.80 aB	4.99 ± 0.33 aB	0.066 ± 0.006 aB
Simpson	T1	0.45 ± 0.005 aA	3.91 ± 0.02 aA	268 ± 1.28 aA	21.35 ± 0.45 aA	5.46 ± 0.10 cA	0.080 ± 0.002 aA
	T2	0.29 ± 0.011 dA	2.91 ± 0.06 cA	232 ± 3.10 cC	18.98 ± 0.72 bA	6.52 ± 0.15 aA	0.082 ± 0.004 aA
	T3	0.32 ± 0.002 cB	3.24 ± 0.01 bB	269 ± 0.63 aA	15.34 ± 0.05 cC	4.74 ± 0.02 dB	0.057 ± 0.000 bC
	T4	0.37 ± 0.003 bA	3.19 ± 0.01 bA	247 ± 0.86 bB	19.15 ± 0.06 bA	6.00 ± 0.02 bB	0.078 ± 0.000 aB
	T5	0.29 ± 0.002 dB	3.30 ± 0.01 bB	253 ± 0.58 bA	15.54 ± 0.09 cB	4.71 ± 0.03 dB	0.061 ± 0.000 bB
Grand Rapids	T1	0.43 ± 0.004 aA	3.81 ± 0.02 aB	261 ± 1.02 aA	20.47 ± 0.14 aA	5.37 ± 0.05 cAB	0.078 ± 0.001 aA
	T2	0.29 ± 0.009 eA	2.81 ± 0.04 cB	248 ± 1.75abB	16.71 ± 0.36 bB	5.94 ± 0.06 bB	0.067 ± 0.002 bB
	T3	0.34 ± 0.002 cA	3.56 ± 0.01 bA	235 ± 0.55 cB	19.92 ± 0.09 aA	5.60 ± 0.02 bcA	0.085 ± 0.001 aA
	T4	0.32 ± 0.002 dB	2.90 ± 0.00 cB	230 ± 0.40 cC	19.65 ± 0.14 aA	6.78 ± 0.04 aA	0.085 ± 0.001 aA
	T5	0.37 ± 0.006 bA	3.62 ± 0.03 aB	241 ± 1.36 bcB	20.33 ± 0.12 aA	5.62 ± 0.06 bcA	0.084 ± 0.001 aA

Mean and standard error (SE, n = 5); CV = coefficient of variation; Means followed by equal lowercase letters in the column do not differ for the interaction of treatments within each cultivar by Tukey’s test (*p* ≤ 0.05). Means followed by equal capital letters in the column do not differ for the interaction of cultivars within each treatment by Tukey’s test (*p* ≤ 0.05). T1—irrigation with low salinity water (0.53 dS m^−1^, control); T2—irrigation with high salinity water (4.0 dS m^−1^, saline stress); T3—saline stress + exogenous application of ascorbic acid (50 µM L^−1^); T4—saline stress + exogenous application of gibberellic acid (50 µM L^−1^); and T5—saline stress + exogenous application of salicylic acid (50 µM L^−1^).

**Table 2:** Summary of F test and test of means for maximum PSII quantum efficiency (*Fv*/*Fm*), PSII quantum efficiency (*Y*), minimum fluorescence of illuminated plant tissue (*Fo*’), photochemical extinction coefficient (*qL*), regulated photochemical extinction quantum yield (*Y_NPQ_*), and unregulated photochemical extinction quantum yield (*Y_NO_*) of lettuce cultivars irrigated with saline water submitted to exogenous application of organic acids at 30 days after transplanting.

F Test (*p*-Value)						
Variation Sources	*Fv*/*Fm*	*Y*	*Fo*’	*qL*	*Y_NPQ_*	*Y_NO_*
Block	0.5311	0.3055	0.2092	0.2165	0.4178	0.1908
Treatments (T)	0.0138	0.0000	0.0748	0.0367	0.0000	0.0002
Cultivars (C)	0.0406	0.6369	0.0446	0.1561	0.4537	0.9793
T × C	0.8372	0.5320	0.9427	0.6956	0.4998	0.7469
CV (%)	1.65	10.27	20.42	41.98	17.06	31.52
Tukey’s Test (Mean ± SE)						
Treatments	*Fv*/*Fm*	*Y*	*Fo’*	*qL*	*Y_NPQ_*	*Y_NO_*
T1	0.769 ± 0.003 ab	0.701 ± 0.006 a	2.40 ± 0.141 b	0.019 ± 0.002 ab	0.251 ± 0.005 d	0.048 ± 0.003 b
T2	0.775 ± 0.002 ab	0.637 ± 0.008 ab	2.98 ± 0.155 a	0.022 ± 0.002 a	0.318 ± 0.008 bc	0.045 ± 0.002 b
T3	0.776 ± 0.004 ab	0.602 ± 0.011 b	2.74 ± 0.187 ab	0.017 ± 0.002 ab	0.343 ± 0.011 b	0.054 ± 0.004 ab
T4	0.764 ± 0.005 b	0.531 ± 0.032 c	2.66 ± 0.108 ab	0.013 ± 0.002 b	0.400 ± 0.026 a	0.070 ± 0.007 a
T5	0.780 ± 0.001 a	0.678 ± 0.010 a	2.58 ± 0.104 ab	0.020 ± 0.002 ab	0.277 ± 0.009 cd	0.044 ± 0.002 b
Cultivars	*Fv*/*Fm*	*Y*	*Fo’*	*qL*	*Y_NPQ_*	*Y_NO_*
SVR 2005	0.767 ± 0.003 A	0.621 ± 0.021 A	2.90 ± 0.123 A	0.020 ± 0.002 A	0.328 ± 0.018 A	0.052 ± 0.005 A
Simpson	0.776 ± 0.003 A	0.632 ± 0.016 A	2.55 ± 0.096 A	0.016 ± 0.001 A	0.315 ± 0.015 A	0.052 ± 0.003 A
Grand Rapids	0.775 ± 0.002 A	0.638 ± 0.014 A	2.57 ± 0.111 A	0.017 ± 0.002 A	0.309 ± 0.012 A	0.053 ± 0.004 A

Mean and standard error (SE, n = 15 for treatments; SE, n = 25 for cultivars); CV = coefficient of variation; Means followed by equal lowercase letters in the column do not differ for treatments and means followed by equal capital letters in the column do not differ for cultivars by Tukey’s test (*p* ≤ 0.05). T1—irrigation with low salinity water (0.53 dS m^−1^, control); T2—irrigation with high salinity water (4.0 dS m^−1^, saline stress); T3—saline stress + exogenous application of ascorbic acid (50 µM L^−1^); T4—saline stress + exogenous application of gibberellic acid (50 µM L^−1^); and T5—saline stress + exogenous application of salicylic acid (50 µM L^−1^).

**Table 3:** Summary of the F test and test of means for electron transport rate (*ETR*) and leaf temperature (*Tl*), in °C, of lettuce cultivars irrigated with saline water submitted to exogenous application of organic acids at 30 days after transplanting.

F Test (*p*-Value)			
Variation Sources		*ETR*	*Tl*
Block		0.0435	0.4102
Treatments (T)		0.0000	0.0000
Cultivars (C)		0.1096	0.0538
T × C		0.0000	0.0000
CV (%)		16.43	0.35
Tukey’s Test (Mean ± SE)			
Cultivars	Treatments	*ETR*	*Tl*
SVR 2005	T1	27.44 ± 1.43 bA	33.90 ± 0.001 bB
	T2	78.18 ± 2.89 aA	33.06 ± 0.024 cB
	T3	75.16 ± 4.30 aA	34.08 ± 0.020 abB
	T4	39.20 ± 6.94 bA	34.16 ± 0.024 aA
	T5	39.60 ± 7.32 bB	34.22 ± 0.196 aB
Simpson	T1	24.82 ± 1.14 cA	34.18 ± 0.020 bA
	T2	61.64 ± 4.93 aB	33.14 ± 0.024 eB
	T3	68.52 ± 4.74 aA	33.38 ± 0.020 dC
	T4	42.18 ± 3.88 bA	33.90 ± 0.001 cB
	T5	63.42 ± 2.76 aA	34.60 ± 0.001 aA
Grand Rapids	T1	25.10 ± 1.53 cA	33.90 ± 0.001 bB
	T2	65.58 ± 3.58 aAB	33.34 ± 0.024 dA
	T3	75.90 ± 1.33 aA	34.32 ± 0.020 aA
	T4	46.14 ± 3.22 bA	33.60 ± 0.001 cC
	T5	25.56 ± 1.02 cC	33.84 ± 0.024 bC

Mean and standard error (SE, n = 5); CV = coefficient of variation; Means followed by equal lowercase letters in the column do not differ for the interaction of treatments within each cultivar by Tukey’s test (*p* ≤ 0.05). Means followed by equal capital letters in the column do not differ for the interaction of cultivars within each treatment by Tukey’s test (*p* ≤ 0.05). T1—irrigation with low salinity water (0.53 dS m^−1^, control); T2—irrigation with high salinity water (4.0 dS m^−1^, saline stress); T3—saline stress + exogenous application of ascorbic acid (50 µM L^−1^); T4—saline stress + exogenous application of gibberellic acid (50 µM L^−1^); and T5—saline stress + exogenous application of salicylic acid (50 µM L^−1^).

**Table 4:** Summary of the F test and test of means for production (*PROD*), number of leaves (*NL*) and shoot dry mass (*SDM*) of lettuce cultivars irrigated with saline water submitted to exogenous application of organic acids at 30 days after transplanting.

F Test (*p*-Value)				
Variation Sources		*PROD* (g)	*NL*	*SDM* (g)
Block		0.0980	0.3531	0.6833
Treatments (T)		0.0000	0.0000	0.0000
Cultivars (C)		0.0466	0.0000	0.0000
T × C		0.0000	0.0000	0.0291
CV (%)		7.26	8.35	14.16
Tukey’s Test (Mean ± SE)				
Cultivars	Treatments	*PROD* (g)	*NL*	*SDM* (g)
SVR 2005	T1	157.77 ± 9.43 aA	16.60 ± 0.748 aA	4.95 ± 0.595 abB
	T2	111.18 ± 6.87 bAB	14.00 ± 0.775 aB	4.37 ± 0.649 abB
	T3	103.72 ± 4.35 bA	14.60 ± 0.245 aC	5.23 ± 0.627 aA
	T4	43.76 ± 4.05 cB	16.60 ± 0.400 aB	2.18 ± 0.135 cB
	T5	115.38 ± 7.81 bAB	14.20 ± 1.871 aB	3.88 ± 0.463 bB
Simpson	T1	154.90 ± 7.90 aA	26.00 ± 1.860 aB	6.08 ± 0.692 aA
	T2	100.79 ± 2.44 cB	15.80 ± 0.490 cB	5.51 ± 0.252 aA
	T3	90.75 ± 3.60 cB	20.20 ± 0.583 bA	5.32 ± 0.416 aA
	T4	40.97 ± 3.79 dB	16.80 ± 0.490 cB	2.12 ± 0.395 bB
	T5	125.84 ± 7.40 bA	21.80 ± 1.897 bA	5.40 ± 0.527 aA
Grand Rapids	T1	123.58 ± 12.63 aB	18.00 ± 1.000 abA	6.38 ± 0.431 aA
	T2	113.85 ± 3.42 abA	20.60 ± 0.812 aA	5.60 ± 0.302 abA
	T3	103.20 ± 2.42 bA	17.20 ± 0.200 bB	5.94 ± 0.313 aA
	T4	60.73 ± 4.65 cA	20.40 ± 0.166 aA	3.48 ± 0.422 cA
	T5	104.16 ± 8.19 bB	20.20 ± 1.327 aA	4.55 ± 0.351 bcAB

Mean and standard error (SE, n = 5); CV = coefficient of variation; Means followed by equal lowercase letters in the column do not differ for the interaction of treatments within each cultivar by Tukey’s test (*p* ≤ 0.05). Means followed by equal capital letters in the column do not differ for the interaction of cultivars within each treatment by Tukey’s test (*p* ≤ 0.05). T1—irrigation with low salinity water (0.53 dS m^−1^, control); T2—irrigation with high salinity water (4.0 dS m^−1^, saline stress); T3—saline stress + exogenous application of ascorbic acid (50 µM L^−1^); T4—saline stress + exogenous application of gibberellic acid (50 µM L^−1^); and T5—saline stress + exogenous application of salicylic acid (50 µM L^−1^).

**Table 5:** Summary of the F test and test of means for potassium (K^+^) content, sodium (Na^+^) content, and sodium/potassium ratio (Na^+^/K^+^) of lettuce cultivars irrigated with saline water submitted to exogenous application of organic acids at 30 days after transplanting.

F Test (*p*-Value)				
Variation Sources	TWC (%)	K^+^	Na^+^	Na^+^/K^+^
Block	0.9703	0.1387	0.3917	0.1260
Treatments (T)	0.0000	0.7281	0.0000	0.0000
Cultivars (C)	0.0000	0.0745	0.0106	0.2751
T × C	0.1950	0.2756	0.0853	0.1885
CV (%)	0.82	24.21	20.56	13.36
Tukey’s Test (Mean ± SE)				
Treatments	TWC (%)	K^+^	Na^+^	Na^+^/K^+^
T1	95.92 ± 0.29 a	45.36 ± 3.40 a	11.53 ± 1.53 b	0.26 ± 0.032 b
T2	95.21 ± 0.28 ab	48.10 ± 4.29 a	25.58 ± 1.75 a	0.54 ± 0.043 a
T3	94.43 ± 0.32 b	46.25 ± 3.25 a	26.56 ± 1.84 a	0.58 ± 0.026 a
T4	94.70 ± 0.28 b	42.47 ± 3.28 a	22.32 ± 1.42 a	0.54 ± 0.025 a
T5	95.98 ± 0.31 a	45.76 ± 3.21 a	23.85 ± 1.43 a	0.53 ± 0.032 a
Cultivars	TWC (%)	K^+^	Na^+^	Na^+^/K^+^
SVR 2005	95.88 ± 0.22 A	45.35 ± 2.56 A	22.23 ± 1.23 A	0.51 ± 0.021 A
Simpson	95.05 ± 0.14 B	49.34 ± 2.70 A	23.84 ± 1.35 A	0.48 ± 0.016 A
Grand Rapids	94.82 ± 0.14 B	42.07 ± 2.79 A	19.85 ± 1.20 B	0.48 ± 0.034 A

Mean and standard error (SE, n = 15 for treatments; SE, n = 25 for cultivars); CV = coefficient of variation; Means followed by equal lowercase letters in the column do not differ for treatments and means followed by equal capital letters in the column do not differ for cultivars by Tukey’s test (*p* ≤ 0.05). T1—irrigation with low salinity water (0.53 dS m^−1^, control); T2—irrigation with high salinity water (4.0 dS m^−1^, saline stress); T3—saline stress + exogenous application of ascorbic acid (50 µM L^−1^); T4—saline stress + exogenous application of gibberellic acid (50 µM L^−1^); and T5—saline stress + exogenous application of salicylic acid (50 µM L^−1^).

**Table 6:** Chemical and physical analysis of the soil used in the experiment.

pH	OM		P	K^+^	Na^+^	Ca^2+^	Mg^2+^	Al^3+^	H+Al	CEC	BS	ESP
	(%)		------(mg dm^−3^)--------			-----------------------(cmol_c_ dm^−3^)----------------------------					------%----	
5.4	2.13		2.0	61.1	16.7	1.6	1.10	0.20	0.33	3.2	63	1.0
ECsedS m^−1^		SDkg dm^−3^		Sand				Silt		Clay		
				----------------------------------------(g kg^−1^)----------------------------------------								
0.58		1.60		820				30		150		

pH—Potential hydrogen; OM—Organic matter; ECse—Electrical conductivity of the saturation extract of the soil; SD—Soil density; CEC—Cation exchange capacity; BS—Base saturation; ESP—Exchangeable sodium percentage.

**Table 7:** Physicochemical characterization of the water sources used in the experiment.

Water Source	pH	EC	K^+^	Na^+^	Mg^2+^	Ca^2+^	Cl^−^	CO_3_^2−^	HCO_3_^−^	SAR
		dS m^−1^	mmol_c_ L^−1^							(mmol_c_ L^−1^)^0.5^
1	7.57	0.53	0.31	3.79	1.20	0.83	2.40	0.60	3.20	3.76
2	7.10	9.50	0.83	54.13	24.20	37.8	116.0	0.00	3.40	9.70

Water source 1—supply water; Water source 2—reject brine; SAR—Sodium adsorption ratio.

**Table 8:** Electrical conductivity (ECse) and pHse of the saturation extract of soil cultivated with lettuce as a function of irrigation with saline water and application of organic acids.

Soil Salinity	T1	T2	T3	T4	T5
ECse (dS m^−1^)	2.0	6.0	5.0	6.1	5.5
pHes	7.0	7.8	7.2	7.7	7.5

T1—irrigation with low salinity water (0.53 dS m^−1^, control); T2—irrigation with high salinity water (4.0 dS m^−1^, saline stress); T3—saline stress + exogenous application of ascorbic acid (50 µM L^−1^); T4—saline stress + exogenous application of gibberellic acid (50 µM L^−1^); and T5—saline stress + exogenous application of salicylic acid (50 µM L^−1^).

## Data Availability

All data are presented in the paper.
